# Identification of Jasmonic Acid Biosynthetic Genes in Sweet Cherry and Expression Analysis in Four Ancient Varieties from Tuscany

**DOI:** 10.3390/ijms20143569

**Published:** 2019-07-22

**Authors:** Roberto Berni, Giampiero Cai, Xuan Xu, Jean-Francois Hausman, Gea Guerriero

**Affiliations:** 1 University of Siena, Department of Life Sciences, via P.A. Mattioli 4, 53100 Siena, Italy; 2 Trees and timber institute-National research council of Italy (CNR-IVALSA), via Aurelia 49, 58022 Follonica (GR), Italy; 3 Research and Innovation Department, Luxembourg Institute of Science and Technology, 5 avenue des Hauts-Fourneaux, L-4362 Esch/Alzette, Luxembourg

**Keywords:** *Prunus avium*, Tuscan varieties, jasmonic acid, lipoxygenase, bioinformatics, gene expression

## Abstract

Sweet cherries are non-climacteric fruits whose early development is characterized by high levels of the phytohormone jasmonic acid (JA). Important parameters, such as firmness and susceptibility to cracking, can be affected by pre- and postharvest treatments of sweet cherries with JA. Despite the impact of JA on sweet cherry development and fruit characteristics, there are no studies (to the best of our knowledge) identifying the genes involved in the JA biosynthetic pathway in this species. We herein identify the sweet cherry members of the lipoxygenase family (*13-LOX*); allene oxide synthase, allene oxide cyclase and 12-oxo-phytodienoic acid reductase 3, as well as genes encoding the transcriptional master regulator MYC2. We analyze their expression pattern in four non-commercial Tuscan varieties (‘Carlotta’, ‘Maggiola’, ‘Morellona’, ‘Crognola’) having different levels of bioactives (namely phenolics). The highest differences are found in two genes encoding 13-LOX in the variety ‘Maggiola’ and one MYC2 isoform in ‘Morellona’. No statistically-significant variations are instead present in the allene oxide synthase, allene oxide cyclase and 12-oxo-phytodienoic acid reductase 3. Our data pave the way to follow-up studies on the JA signaling pathway in these ancient varieties, for example in relation to development and post-harvest storage.

## 1. Introduction

*Prunus avium* L. is an economically relevant tree of the *Rosaceae* family producing stone fruits. Its fruits are appreciated worldwide for their aroma, taste and richness in bioactive molecules. Sweet cherries are indeed rich in polyphenols and pentacyclic triterpenes [1,2]. They have a low content of calories. They are cholesterol-free and therefore valuable from a nutraceutical point of view. The ripening of sweet cherries is accompanied by an early increase in endogenous jasmonic acid (JA) levels [3]. This phytohormone is known to regulate different aspects of plant growth and development [4], as well as the response to exogenous stresses [5,6]. 

Given the physiological relevance of JA, many studies in the literature have specifically looked at the response of plants to this phytohormone in relation to flower and seed development [7], fruit ripening [8,9], senescence [10] and secondary metabolite production [11,12,13]. Concerning fruits, the majority of papers have dealt with the postharvest application of JA and its derivatives, which improved shelf life by protecting against pathogen attack and by enhancing the antioxidant content [14]. Studies have also investigated the role of JA in the ripening of climacteric and, to a lesser extent, in non-climacteric fruits: In the former (e.g., apples), jasmonates act with ethylene in regulating ripening [15], while in the latter (e.g., strawberries) the phytohormone affects cell walls, modifying enyzmes, anthocyanin accumulation and lignin biosynthetic genes [16]. Some studies are present in the literature concerning the response of sweet cherries to JA: More specifically, derivatives of this phytohormones were applied both pre- and postharvest to improve the content of bioactives and the firmness of the fruits [8,17,18]. The preharvest application of methyl jasmonate (MeJA) at a concentration of 10 mM improved fruit flesh firmness, slowed down color changes and delayed harvest by one week [8]. Additionally, MeJA (applied at a concentration of 0.4 mM) alone or in combination with 0.1 mM abscisic acid (ABA) significantly reduced the susceptibility to cracking after tests consisting of 6 h-water immersion [17]. Interestingly, this effect was observed over 2 consecutive years and on fruits harvested both at the stage of fruit set and color change. Postharvest treatment of sweet cherries with MeJA also increased the activities of a peroxidase and β-1,3-glucanase: Those findings suggest a possible protection against biotic stress, although little inhibitory activity was observed on *Monilinia fructicola* [18].

Despite the economic value of sweet cherries and the physiological relevance of JA and its derivatives, no study is available, to the best of our knowledge, on the identification of genes related to its biosynthesis and signaling *in P. avium*. The content of JA is known to be extremely low in sweet cherries at maturity [3]; therefore, we made the choice of focusing on JA-related genes in this study. Here we identify the sweet cherry members of the lipoxygenase family (*13-LOX*), *allene oxide synthase* (*AOS*), *allene oxide cyclase* (*AOC*) and *12-oxo-phytodienoic acid reductase 3* (*OPR3*), as well as the transcription factor (TF) *MYC2*. We provide data concerning gene expression in non-commercial ancient fruits collected at maturity (60 days post anthesis, dpa) and belonging to the regional germplasm of Tuscany [19]. Since no similar study exists on *P. avium* (and even more so on these ancient fruits) and since maturity is the interesting stage for an exploitation, we decided to measure gene expression at commercial harvest. In particular, we focused our attention on 4 varieties which were previously described to contain varying levels of bioactives (‘Carlotta’ and ‘Maggiola’ that produce lower amounts of phenolics, ‘Morellona’ and ‘Crognola’ that are instead high producers) [1]. The goal is to pave the way to follow-up studies aiming at analyzing JA signaling in these ancient fruits; e.g., both during development and postharvest storage. A kinetic study could indeed provide additional insight into the relationship existing between JA biosynthesis and the content of phenolics in sweet cherries and ultimately unveil potential links between early development and the higher antioxidant capacities measured in some of the Tuscan varieties. A study on postharvest treatments with the phytohormone or its derivatives will evaluate the effects on the stability of the fruits produced by the varieties studied here. A high level of bioactives, together with an increase in postharvest stability are indeed important characteristics for a potential exploitation of these ancient fruits. 

## 2. Results and Discussion

### 2.1. Identification of JA Biosynthetic Genes in Sweet Cherries

**Figure 1:**
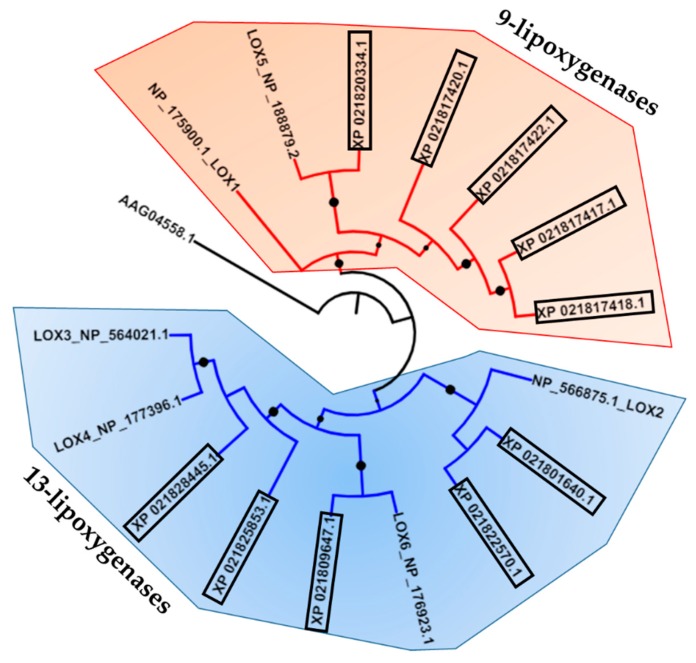
Maximum likelihood phylogenetic tree (number of bootstraps in ultrafast mode: 1000) of different LOX protein’s full length sequences from thale cress and *P. avium* (boxed). Accession numbers from thale cress are NP_175900.1, NP_188879.2, NP_566875.1, NP_176923.1, NP_177396.1, NP_564021.1. All the other accessions in the tree are sequences from *P. avium* (see Table 1). Bootstrap values comprised of between 0.8 and 1 are displayed (the bigger the circle, the higher the bootstrap value). The tree was rooted with *Pseudomonas aeruginosa* lipoxygenase PAO1 (accession number AAG04558.1).

The BLAST analysis carried out to mine the *13-LOX* genes in the genome of sweet cherry [20] using the six known thale cress sequences [21] led to the identification of five *9-LOX*s and six members of the *13-LOX* family. This number falls in the same range as the total number of LOX found in *Carica papaya* and *Vitis vinifera*, where a total of 11 and 13 genes encoding LOXs were reported [22]. The maximum likelihood phylogenetic analysis showed that *P. avium*’s corresponding proteins nicely cluster into two separate branches, one for 9-LOXs (Pav_sc0000700.1_g070.1.mk/XP_021817420.1, Pav_sc0000700.1_g050.1.mk/XP_021817418.1, Pav_sc0000700.1_g080.1.mk/XP_021817422.1, Pav_sc0000861.1_g060.1.mk/XP_021820334.1, Pav_sc0000648.1_g280.1.mk/XP_021817417.1) and one for 13-LOXs (Figure 1). 

Based on the phylogenetic relationship with the thale cress 13-LOX, we could assign a nomenclature for the identified sweet cherry lipoxygenases (Table 1). The gene *PavLOX2.3* corresponds to two paralogs; however, it should be noted that Pav_sc0001040.1_g230.1.br is much shorter and may represent an incomplete sequence; indeed the best hit in NCBI corresponds to XP_021822570.1, which is 914 amino acids. The presence of a chloroplast signaling peptide was confirmed for all of the 13-LOX from *P. avium* (scores berween 0.509–0.565). 

Additionally, we identified two *AOC*s, one *AOS* and one thale cress ortholog of *OPR3* (Table 1). Interestingly, three orthologs of the master regulator *MYC2* were retrieved from the genome of sweet cherry (Table 1): The gene named *MYC2.2* showed the highest percentage identity (obtained when using the blastx algorithm, and as input, the GDR nucleotide sequences indicated in Table 1) with *Arabidopsis* MYC2 (85.71%), while MYC2 and MYC2.3 had lower scores (37.88% and 52.51%, respectively). 

**Figure 2:**
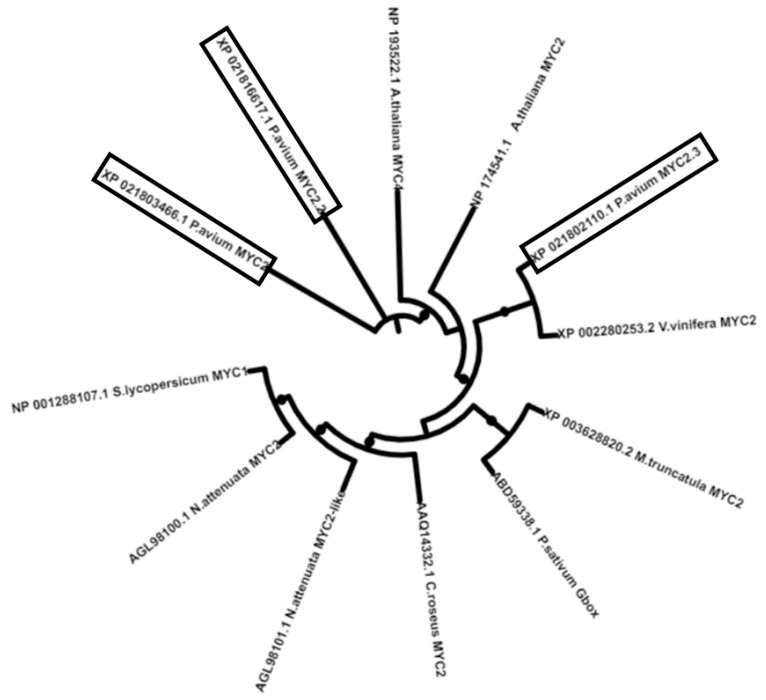
Unrooted maximum likelihood phylogenetic tree (number of bootstraps in ultrafast mode: 1000) of different MYC2 protein’s full length sequences from *P. avium* (boxed) and other species taken from [23]. Bootstrap values comprised of between 0.8 and 1 are displayed (the bigger the circle, the higher the bootstrap value).

The MYC2 phylogenetic tree shows that PavMYC2.3 branches together with *V. vinifera* MYC2 (Figure 2), while the other sweet cherry MYC2 proteins do not show obvious similarities with MYC2 from other plant species [23].

### 2.2. Total and Targeted Quantification of Phenolics in the Four Ancient Varieties

We previously characterized six ancient Tuscan varieties of *P. avium* sampled in 2017 from a nutraceutical point of view and ranked them according to their content in antioxidants, polyphenols, flavonoids and anthocyanins [1]. We here quantify the same parameters in a subset of four varieties known to show differences in the content of bioactives and sampled in the year 2018. We indeed wished to confirm the stability in the trend of the measured parameters over a different year of harvest. The results are shown in Table 2: Concerning the total antioxidants, the variety ‘Crognola’ ranks first, followed by ‘Morellona’, while ‘Maggiola’ is the lowest. The same ranking is observed for the polyphenols and flavonoids, while anthocyanins are highest in ‘Morellona’. It should be noted that this variety is red fleshed, differently from all the others. Some differences are, however, observed with respect to the previously published data on fruits sampled in 2017 [1], where ‘Carlotta’ was the lowest producer of antioxidants, polyphenols and anthocyanins. In 2017, the highest amount of anthocyanins was observed for ‘Crognola,’ despite the absence of a red flesh.

The targeted quantification of phenolics on the cherries sampled in 2018 reflects the spectrophotometric analyses, with the only exception of *p*-coumaric acid, whose abundance is significantly higher in ‘Maggiola’ as compared to ‘Carlotta’ and even ‘Morellona’ (Table 3). Despite being fruits sampled in an experimental field, the standard devations are overall low: This is partly due to our choice of pooling several fruits per biological replicate and to the grinding of the whole tissues (exocarp, mesocarp and skin). Eventual differences in the content of bioactive molecules in the pericarp and skin are thus levelled across replicates. These results differ from the values reported in 2017, where the values were the lowest for ‘Maggiola’ [1]. The samples were harvested from trees grown in an experimental field and therefore exposed to the natural environment—the differences observed may have been due to the varying climatic conditions between 2017 and 2018. It will be interesting to carry out high-throughput studies involving proteomics and transcriptomics to understand the reasons for the recorded differences in the two years of harvest. 

### 2.3. Gene Expression Analysis of JA Biosynthetic Genes in the Four Ancient Varieties

**Figure 3:**
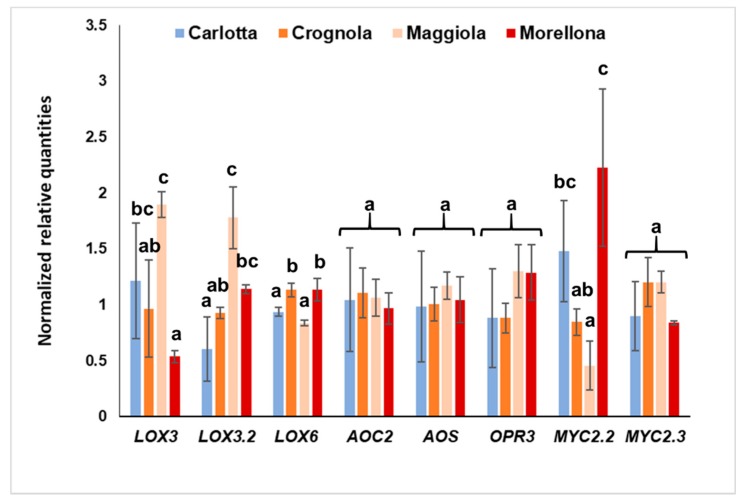
Relative expression of JA biosynthetic genes in the four Tuscan varieties. Error bars refer to the standard deviation (number of independent biological replicates = 4). Different letters indicate statistically significant differences among the groups of the one-way ANOVA with Tukey’s post-hoc test.

The qPCR analysis was performed on the same four varieties and on 8 targets whose expression was detectable in mature fruits, namely *LOX6*, *AOC2*, *AOS*, *LOX3*, *LOX3.2*, *MYC2.2*, *MYC2.3* and *OPR3*. We reasoned that these four varieties would be the best samples to highlight an eventual correlation between bioactive content and the expression of genes related to JA biosynthesis. As can be seen in Figure 3, the four ancient varieties differ in the expression of *LOX* genes and *MYC2.2*. More specifically, the variety ‘Maggiola’ shows the highest expression of *LOX3* and *LOX3.2. LOX*s code for non-haem iron-containing dioxygenases catalyzing the oxygenation of polyunsaturated fatty acids (PUFAs), such as α-linoleic or α-linolenic acid, and lead to the synthesis of molecules collectively known as oxylipins [24]. LOXs known as 13-LOXs catalyze the oxygenation of the C atom in position 13 in the PUFA α-linolenic acid which will then enter the JA biosynthetic pathway via the action of the enzymes AOS and AOC [25]. The higher expression of *LOX3* and *LOX3.2* in ‘Maggiola,’ coupled to the lack of any statistically significant differences in *AOC2* and *AOS* expressions in the Tuscan fruits, suggest that the identified genes partake in other biosynthetic branches (at least in the developmental stage herein analyzed). For example, the two genes may be involved in the LOX branch linked with hydroperoxide lyases (HPLs) which leads to the formation of volatiles responsible for the aroma of fruits. Plant HPLs have been classified into those acting on 13-hydroperoxylinoleic acid and 13-hydroperoxy-α-linolenic acid and those cleaving 9-hydroperoxy isomers of linoleic and α-linolenic acids [26]. In the literature, it was indeed shown that, at maturity, the content of C6 aldehydes increased in Chinese varieties of sweet cherry [27].

*LOX6* shows a different pattern (Figure 3): Even if slight, the higher expression in ‘Crognola’ and ‘Morellona’ is statistically significant and follows the same trend observed for the antioxidants, polyphenols, flavonoids and anthocyanins (Table 2). The Pearson correlation analysis showed a strong positive correlation between *LOX6* and total antioxidanty, polyphenols, flavonoids, and anthocyanins, as well as chlorogenic acid, catechin and cyanidin-3-glucoside (Supplementary File 1). Although JA is known to activate the late genes in the anthocyanin biosynthetic pathway via COI1 (Coronatine-Insensitive 1) in thale cress [28], a previous study comparing apple and sweet cherry fruits highlighted, in the latter, a lack of correlation between endogenous JA/MeJA content and anthocyanin accumulation at maturity [3]. Further experimental proofs are needed to understand the role of *P. avium* LOX6. 

We also measured the expression of two genes encoding members of the master regulator MYC2: *MYC2.2* and *MYC2.3*, which show different trends, with the former expressed at the highest levels in ‘Morellona’ and the latter displaying no significant variations (Figure 3). MYC2 is known to be involved in flavonoid biosynthesis [29,30] and ‘Morellona’ is one of the highest producers; the gene is, however, expressed at lower levels in ‘Crognola’, despite its high levels of bioactives (Table 2 and Table 3). RNA was extracted from samples comprising both the skin and mesocarp. The variety ‘Crognola’ is characterized by fruits displaying an intense red-colored skin but a yellow pulp: This may be due to a lower expression of *MYC2.2* with respect to ‘Morellona’. 

## 3. Materials and Methods

### 3.1. Fruit Harvesting

The 18-year-old sweet cherry trees were grown in an experimental field of the National Research Council (CNR) in Follonica (Tuscany, Italy). The coordinates of the experimental field were previously reported [31]. The fruits were harvested by picking them all over the tree canopy at an average height of 1.70–1.90 m from the ground. Sampling took place in 2018 (sampling day: 18 May 2018, sampling time: Between 9:00 and 10:00 a.m., daily maximum temperature: 24 °C, daily minimum temperature: 8 °C, humidity: 74%). The sampled fruits were immediately placed in liquid nitrogen, then brought to the laboratory for long term storage at −80 °C. 

### 3.2. Chemical Assays, HPLC Analyses and Statistics

For the determination of the total antioxidants, polyphenols, flavonoids and anthocyanins, we followed the methods previously described [1]. The analyses were performed in 3 technical replicates and 3 biological replicates (each biological replicate consisted of a pool of 3 sweet cherry fruits). The quantification of targeted secondary metabolites was performed on an HPLC-DAD system (PerkinElmer Series 200 HPLC Systems, Diode Array Detector, Norwalk, CT, USA) and by using curves generated with standard molecules, as previously reported [1]. The values obtained by averaging 3 independent technical replicates were log-transformed, and a one-way ANOVA with a Tukey’s post-hoc test was performed with IBM SPSS Statistics v19 (IBM SPSS, Chicago, IL, USA) to determine the statistically significant differences among groups. 

### 3.3. RNA Extraction, Primer Design and Real-Time PCR Data Analysis

The RNA extraction method, together with quantification, assessment of integrities, reverse transcription and real-time PCR conditions were as previously reported [31]. The primers used for the reference genes have been previously published [2,31]; those for the JA biosynthetic genes are reported in Table 4. They were designed with Primer3Plus (http://www.bioinformatics.nl/cgi-bin/primer3plus/primer3plus.cgi, last accessed on: 20 May 2019) and the presence of self-dimers, hetero-dimers, hairpins was checked with OligoAnalyzer 3.1 (http://eu.idtdna.com/calc/analyzer, last accessed on: 20 May 2019).

The gene expression values were determined with qBase^PLUS^ (version 3.2, Biogazelle, Ghent, Belgium) by using *actin7* and *PP2A* as reference genes. Statistics were performed with IBM SPSS Statistics v19. A one-way ANOVA with a Tukey’s post-hoc test was carried out after having transformed the NRQs (Normalized Relative Quantities) in log2 values and after having confirmed the normal distribution with a quantile-quantile plot (Q-Q plot). Four independent biological replicates, each consisting of a pool of 3 fruits were used for the gene expression study.

### 3.4. Bioinformatics

Sweet cherry LOX, MYC, AOS, AOC, OPD3 were obtained by blasting the thale cress protein sequences in NCBI and the GDR (Genome Data for *Rosaceae*) database (https://www.rosaceae.org/blast/nucleotide/protein, last accessed on: 20 May 2019). The phylogenetic analysis was carried out by using the full-length LOX and MYC protein sequences identified in sweet cherry and thale cress, as well as other plant species. The pair-wise multiple alignment of LOX and MYC2 proteins from *P. avium*, *Arabidopsis thaliana* and the other plant species indicated in Figure 1 and Figure 2 was performed with CLUSTAL-Ω (http://www.ebi.ac.uk/Tools/msa/clustalo/, last accessed on: 20 May 2019) [32], and the alignment was then used for the maximum likelihood phylogenetic tree using the online program W-IQ-TREE [33] (bootstraps in ultrafast mode: 1000; in auto mode, without selecting FreeRate Heterogeneity), available at http://iqtree.cibiv.univie.ac.at. The tree was visualized with iTOL (available at https://itol.embl.de/, last accessed on: 20 May 2019). 

The chloroplast targeting sequence was verified with ChloroP [34] (available at http://www.cbs.dtu.dk/services/ChloroP/, last accessed on: 20 May 2019). 

## 4. Conclusions

We have herein identified the JA biosynthetic genes in the economically relevant fruit tree *P. avium*, and measured their expression in four ancient varieties from Tuscany characterized by different levels of bioactives. The data suggest that *LOX3.2* in ‘Maggiola’ fruits at maturity is not linked to JA production, but to other biochemical branches; e.g., volatile production. *MYC2.2* is highest in ‘Morellona,’ a variety ranking first in terms of anthocyanins content. With this Communication, we wish to inspire future studies focusing on kinetics and postharvest treatment with JA (or its derivatives), to evaluate the relationship between bioactive contents and JA production, as well as to assess the stability of the Tuscan fruits during postharvest storage.

## Figures and Tables

**Table 1:** Details of the identified JA biosynthetic genes (*13-LOX*, *AOS*, *AOC*, *OPR3*, *MYC2*) in *P. avium*, with proposed nomenclature, GDR (Genomic Database for *Rosaceae*) codes and NCBI accession numbers. Genes separated by/indicate paralogs.

Proposed Nomenclature	GDR Code	NCBI Accession (Best Hit)
PavLOX2	Pav_sc0001040.1_g410.1.mk	XP_021822553.1
PavLOX2.2	Pav_sc0001040.1_g480.1.mk	XP_021822570.1
PavLOX2.3	Pav_sc0005842.1_g020.1.mk/Pav_sc0001040.1_g230.1.br	XP_021801640.1/XP_021822570.1
PavLOX3	Pav_sc0001305.1_g640.1.mk	XP_021825853.1
PavLOX3.2	Pav_sc0001580.1_g270.1.mk	XP_021828445.1
PavLOX6	Pav_sc0000351.1_g320.1.mk	XP_021809647.1
PavAOS	Pav_sc0000890.1_g1300.1.mk/Pav_sc0004356.1_g050.1.mk	XP_021820947.1/XP_021800682.1
PavAOC	Pav_sc0000618.1_g380.1.mk	XP_021804158.1
PavAOC 2	Pav_sc0000567.1_g750.1.mk	XP_021814377.1
PavOPR3	Pav_sc0000129.1_g860.1.mk	XP_021804971.1
PavMYC2	Pav_sc0000107.1_g180.1.mk	XP_021803466.1
PavMYC2.2	Pav_sc0000652.1_g730.1.mk	XP_021816617.1
PavMYC2.3	Pav_sc0006499.1_g050.1.mk	XP_021802110.1

**Table 2:** The table reports the values (± standard deviation, number of independent biological replicates = 3) obtained from the sweet cherry varieties sampled in 2018. The results show the total content of antioxidants expressed as mmol Fe^2+^ per 100 g fresh weight (FW), polyphenols as mg of GAE (gallic acid equivalents) per 100 g of FW, flavonoids as mg of QeE (quercetin equivalents) per 100 g of FW and anthocyanins as CyE (cyanidin-3-glucoside equivalents) per 100 g of FW. Different letters indicate statistically significant differences (*p* < 0.05) among groups at the one-way ANOVA with Tukey’s post-hoc test.

Variety	Total Antioxidants (mmol Fe^2+^ mmol/100g FW)	Polyphenols (mg GAE/100g FW)	Flavonoids (mg QeE/100g FW)	Anthocyanins (mg CyE/100g FW)
‘Carlotta’	1.73 ± 0.01 ^b^	159.18 ± 0.41 ^b^	49.33 ± 0.71 ^b^	34.46 ± 0.81 ^b^
‘Morellona’	2.22 ± 0.02 ^c^	313.11 ± 3.55 ^c^	97.86 ± 1.56 ^c^	65.27 ± 1.03 ^d^
‘Maggiola’	1.43 ± 0.02 ^a^	137.70 ± 2.01 ^a^	40.06 ± 1.23 ^a^	32.41 ± 0.91 ^a^
‘Crognola’	3.07 ± 0.01 ^d^	387.11 ± 1.29 ^d^	102.05 ± 2.42 ^d^	58.46 ± 1.11 ^c^

**Table 3:** The table reports the HPLC quantifications of specific bioactive molecules in the sweet cherry varieties sampled in 2018 expressed as μg per gram of fresh weight (FW). The values are indicated with the relative standard deviation (number of independent biological replicates = 3); different letters indicate statistically significant differences (*p* < 0.05) among groups at the one-way ANOVA with Tukey’s post-hoc test.

Variety	Chlorogenic Acid (μg/g FW)	*p*-Coumaric Acid (μg/g FW)	(+)-Catechin (μg/g FW)	Rutin (μg/g FW)	Cyanidin-3-Glucoside (μg/g FW)
‘Carlotta’	179.42 ± 0.83 ^b^	29.02 ± 0.57 ^a^	122.83 ± 4.96 ^b^	32.54 ± 0.63 ^b^	59.50 ± 1.02 ^b^
‘Morellona’	276.38 ± 0.98 ^c^	51.17 ± 1.11 ^b^	172.55 ± 1.10 ^c^	39.05 ± 0.43 ^b^	80.62 ± 0.77 ^c^
‘Maggiola’	99.23 ± 0.57 ^a^	76.50 ± 0.88 ^c^	46.74 ± 0.99 ^a^	29.11 ± 0.72 ^a^	31.26 ± 1.47 ^a^
‘Crognola’	312.67 ± 1.11 ^d^	123.49 ± 0.64 ^d^	219.44 ± 2.49 ^d^	99.78 ± 0.55 ^c^	149.77 ± 1.29 ^d^

**Table 4:** List of primers with details of the sequences, amplicon sizes and amplification efficiencies.

Name	Sequence (5’→3’)	R^2^	Tm (°C)	Amplicon Size (bp)	Efficiency (%)
Pav_LOX3 Fwd	TCTTGACCTCATTGGGAACC	0.995	79.65	85	104.03
Pav_LOX3 Rev	ACCTGCTTGGATGGTGAATC				
Pav_LOX3.2 Fwd	GCCATCAGTGAAGATTTGGTG	0.995	81.39	106	96.7
Pav_LOX3.2 Rev	CCTCCTTGATCTTGTTCCTCAC				
Pav_LOX6 Fwd	CAGCATGGTGAAAGAGGTTC	0.992	79.51	86	91.51
Pav_LOX6 Rev	AAGCAAATCTATCCCCTTT				
Pav_AOS Fwd	GGAGATGTTGTTCGGGTTTC	0.995	78.98	74	104.42
Pav_AOS Rev	CTACAAACTCCTCCGCT				
Pav_AOC2 Fwd	CAATCTCTCGCATTCCTTCC	0.996	80.32	71	92.44
Pav_AOC2 Rev	AGTTCTTGGAGTTTGGGAA				
Pav_OPR3 Fwd	CAAGTGGTGGAGCATTATCG	0.987	85.01	89	104.42
Pav_OPR3 Rev	AGTTTTGAGCCCCAGTCTTG				
Pav_MYC2.2 Fwd	CCGCTCTGTTGTTCCAAATG	0.982	80.05	106	95.12
Pav_MYC2.2 Rev	TAGCCTCCAATTCCTCAACC				
Pav_MYC2.3 Fwd	GGGTGAAGGGTTTTACAAGG	0.997	83.31	96	91.58
Pav_MYC2.3 Rev	GGACTTTTTTCCTGTACTCC				

## Supplementary Materials

The following are available online at https://www.mdpi.com/1422-0067/20/14/3569/s1.
